# Nanocomposites SnO_2_/SiO_2_:SiO_2_ Impact on the Active Centers and Conductivity Mechanism

**DOI:** 10.3390/ma12213618

**Published:** 2019-11-04

**Authors:** Dayana Gulevich, Marina Rumyantseva, Artem Marikutsa, Tatyana Shatalova, Elizaveta Konstantinova, Evgeny Gerasimov, Alexander Gaskov

**Affiliations:** 1 Chemistry Department, Moscow State University, Moscow 119991, Russia; dayana-nsu@mail.ru (D.G.); artem.marikutsa@gmail.com (A.M.); shatalovatb@gmail.com (T.S.); gaskov@inorg.chem.msu.ru (A.G.); 2 Faculty of Physics, Moscow State University, Moscow 119991, Russia; liza35@mail.ru; 3 National Research Center Kurchatov Institute, Moscow 123182, Russia; 4 Department of Nano-, Bio-, Information Technology and Cognitive Science, Moscow Institute of Physics and Technology, Dolgoprudny, Moscow Region 141701, Russia; 5 Boreskov Institute of Catalysis SB RAS, Novosibirsk, 630090 Russia; gerasimov@catalysis.ru

**Keywords:** nanocomposites, tin dioxide, silicon dioxide, oxygen chemisorption, active surface groups, paramagnetic centers, conductivity mechanism

## Abstract

This paper is focused on the effect of the stabilizing component SiO_2_ on the type and concentration of active sites in SnO_2_/SiO_2_ nanocomposites compared with nanocrystalline SnO_2_. Previously, we found that SnO_2_/SiO_2_ nanocomposites show better sensor characteristics in CO detection (lower detection limit, higher sensor response, and shorter response time) compared to pure SnO_2_ in humid air conditions. Nanocomposites SnO_2_/SiO_2_ synthesized using the hydrothermal method were characterized by low temperature nitrogen adsorption, XRD, energy dispersive X-ray spectroscopy (EDX), thermo-programmed reduction with hydrogen (TPR-H_2_), IR-, and electron-paramagnetic resonance (EPR)-spectroscopy methods. The electrophysical properties of SnO_2_ and SnO_2_/SiO_2_ nanocomposites were studied depending on the oxygen partial pressure in the temperature range of 200–400 °C. The introduction of SiO_2_ results in an increase in the concentration of paramagnetic centers Sn^3+^ and the amount of surface hydroxyl groups and chemisorbed oxygen and leads to a decrease in the negative charge on chemisorbed oxygen species. The temperature dependences of the conductivity of SnO_2_ and SnO_2_/SiO_2_ nanocomposites are linearized in Mott coordinates, which may indicate the contribution of the hopping mechanism with a variable hopping distance over local states.

## 1. Introduction

The development of high-temperature sensors necessary for local monitoring of the concentration of toxic compounds in exhaust (flue) gases and atmospheric emissions requires the creation of new materials to be stable at high temperatures of 300–600 °C. These specific tasks imply a high ambient temperature, which determines the requirements primarily for the stability of materials. This distinguishes high-temperature sensors from other types of semiconductor sensors operating, for example, at room temperature [1,2,3]. The grain growth under a high temperature results in an increase in the area of contact between the crystallites and the formation of necks between the grains. This, in turn, determines the structure and properties of the conducting cluster responsible for the transport of charge carriers. Tin dioxide SnO_2_ is a wide-gap *n*-type semiconductor (Eg = 3.6 eV at 300 K) that has the most widespread technological application as a material for semiconductor gas sensors [4]. In addition to the indicated above low stability of sensor characteristics during long-term functioning at high temperatures, the main disadvantages of the SnO_2_-based sensors are low selectivity and reduced sensitivity in humid air [5]. The increase in the sensitivity and selectivity of nanocrystalline SnO_2_-based materials can be achieved by chemical modification of the surface of tin dioxide [6,7], as well as by using the dynamic temperature mode followed by mathematical processing of the sensor response [8]. However, these approaches also impose additional requirements on the stability of the microstructure of the sensitive material. 

One of the possible ways to solve the problem of low stability of the microstructure at high temperatures is to create nanocomposites based on semiconductor oxides and the stabilizing component, for example amorphous silicon oxide SiO_2_. It was shown that the addition of SiO_2_ allows obtaining composite materials with high specific surface area which demonstrate stable microstructure characteristics during high-temperature annealing [9,10,11,12,13,14,15,16]. An increase in the sensor signal to volatile organic compounds (VOCs) and CO was observed [9,10,11,12,13,14,15,16]. Tricoli et al. demonstrated [9,10] that in SnO_2_/SiO_2_ nanocomposites obtained by direct-flame aerosol deposition, the doping with SiO_2_ prevents SnO_2_ grain and crystal growth, most likely due to formation of interstitial solid solution of Si in the SnO_2_ lattice. It was concluded that SnO_2_/SiO_2_ nanocomposites can enhance the long-term stability and VOC sensitivity of SnO_2_-based gas sensors while having minimal impact on the residual SnO_2_ properties [10]. The SiO_2_@SnO_2_ core–shell nanofibers, composed of amorphous SiO_2_ fiber core, and the outer layer, formed by uniform SnO_2_ particles, were investigated as gas sensors to ethanol, ammonia, benzene, toluene, chloroform, and hexane gases, but exhibited an enhanced gas response to ethanol with a short response time [11]. Similarly, the effects of surface chemical modification with SiO_2_ (using wet-chemical modification through the dehydration-condensation reaction) on the thermal stability and CO gas-sensing properties of SnO_2_ were investigated by Zhan et al. [12]. It was shown that the presence of SiO_2_ on the tin dioxide surface effectively inhibits the growth of SnO_2_ nanocrystals. The sensitivity enhancement in CO detection was ascribed to the ultrafine crystal size, which is less than twice the Debye length. A similar explanation for the increase in sensor sensitivity of SnO_2_-decorated SiO_2_ samples to acetone and ethanol was proposed by Asgari et al. [13]. Information about sensor properties of SnO_2_/SiO_2_ nanocomposites is presented in Table 1. 

At the same time, the addition of SiO_2_ affects not only the microstructure of the SnO_2_ semiconductor matrix, but also the composition of surface-active groups, which alters the reactivity of the obtained materials in the interaction with the gas phase. However, the detailed studies of the effect of SiO_2_ on the surface composition and reactivity of SnO_2_ in the solid-gas interactions are very few. Nalimova et al. [14] demonstrated that the electron beam processing of the sol-gel SnO_2_–SiO_2_ thin films leads to a significant increase in their sensitivity towards acetone and isopropanol vapors. It is found that the observed effect is correlated with an increase in the concentration of the Brønsted acid sites. Gunji et al. [15] studied the gas sensing properties of template synthesized SiO_2_/SnO_2_ core–shell nanofibers towards H_2_ and CO in dry and humid conditions in comparison with SnO_2_ nanoparticles produced by a hydrothermal method. The SiO_2_/SnO_2_ nanofibers showed a prominent sensor response in humid atmosphere. It was supposed that SiO_2_ particles acted as a water absorber to hinder hydroxyl poisoning of adjacent SnO_2_. 

In our previous work [16], the sensor properties of SnO_2_/SiO_2_ nanocomposites obtained by the hydrothermal route were investigated during CO detection in dry and humid (relative humidity RH = 4–65%) air in the temperature range 150–400 °C. It was found that SnO_2_/SiO_2_ nanocomposites show better sensor characteristics in CO detection (lower detection limit, higher sensor response, and shorter response time) compared to pure SnO_2_ in humid air conditions. Moreover, the resistance of SnO_2_/SiO_2_ nanocomposites was less sensitive to the RH change over the whole range of operating temperatures. The obtained sensor parameters of nanocrystalline SnO_2_ and SnO_2_/SiO_2_ nanocomposites [16] are summarized in Table 2. 

This paper analyzes the effect of the stabilizing component SiO_2_ and the appearance of the SnO_2_/SiO_2_ interface on the type and concentration of active sites in SnO_2_/SiO_2_ nanocomposites compared with nanocrystalline SnO_2_. The focus is on the predominant forms of chemisorbed oxygen and paramagnetic centers and their relationship with the mechanism of charge carrier transport in these materials.

## 2. Materials and Methods 

### 2.1. Materials Synthesis

Semiconductor materials based on SnO_2_/SiO_2_ were obtained by hydrothermal processing of a xerogel SnO_2_ ·xH_2_O and an alcohol solution of Si(OH)_4_. SnCl_4_·5H_2_O (98%, Sigma-Aldrich, Saint Louis, MO, USA) and tetraethoxysilane (TEOS) (98%, Sigma-Aldrich) were used as Sn^4+^ and Si^4+^ precursors, respectively. The synthesis process is described in detail in our previous work [16]. In brief, SnO_2_·xH_2_O xerogel was obtained by hydrolysis of 3M SnCl_4_∙5H_2_O aqueous solution with 25% NH_3_·H_2_O aqueous solution, followed by drying at 50 °C. Si(OH)_4_ alcohol solution was produced through TEOS hydrolysis in a reaction medium consisting of 90% ethyl alcohol, 5% water, and 5% TEOS (by volume) at pH = 4. To obtain the SnO_2_/SiO_2_ composites, the SnO_2_·xH_2_O xerogel and Si(OH)_4_ alcohol solution were autoclaved at 150 °C for 24 h with a constant stirring. The reaction product was repeatedly washed with ethyl alcohol and water, dried at room temperature, and annealed at 600 °C for 24 h. The annealing temperature was selected based on the thermal analysis with mass spectral determination of CO_2_ (*m*/*z* = 44). According to the obtained data, all possible organic by-products of the TEOS hydrolysis decomposed at a temperature of 500–550 °C [16]. The designations of samples and their characteristics are given in Table 3.

### 2.2. Materials Characterization

The composition of the samples was investigated by energy dispersive X-ray spectroscopy (EDX) using a Zeiss NVision 40 (Carl Zeiss, Oberkochen, Germany) scanning electron microscope equipped with a X-Max detector (Oxford Instruments, Abington, UK) operated at 20 kV.

The phase composition was determined by X-ray diffraction on a DRON-4 diffractometer (SPE "Burevestnik", Saint-Petersburg, Russia) using monochromatic CuKα radiation (λ = 1.5406 Å). The survey was carried out in the range of 2θ = 10–60 ° with a step of 0.1 °. The crystallite size *d_XRD_* of the SnO_2_ phase was estimated from the broadening of the (110) and (101) reflections using the Scherer formula. Specific surface area *S_BET_*was determined by low-temperature nitrogen adsorption on Chemisorb 2750 (Micromeritics, Norcross, GA, USA) with subsequent analysis using the BET model (single point).

The microstructure of the SnO_2_/SiO_2_ nanocomposites was studied by high-resolution transmission electron microscopy (HRTEM) on a JEM 2010 (JEOL, Tokyo, Japan) instrument with an accelerating voltage of 200 kV and a lattice resolution of 0.14 nm. The images were recorded using a CCD matrix of the Soft Imaging System (Mega View III, Münster, Germany). 

The surface composition (including hydroxyl groups, adsorbed water, and paramagnetic centers) was studied using Fourier transformed infrared spectroscopy (FTIR), thermal analysis, and electron-paramagnetic resonance (EPR) spectroscopy. The IR spectra were recorded on a Frontier FTIR spectrometer (Perkin Elmer Inc., Waltham, MA, USA) in the transmission mode in the range of 4000–400 cm^−1^ with 1 cm^−1^ step. The powders (1 wt%) were grinded with dried KBr (Aldrich, “for FTIR analysis”) and pressed into tablets. Thermal analysis of the samples was carried out on a STA 409 HC Luxx thermal analyzer (Netzsch-Gerätebau GmbH, Selb, Germany). The samples were heated in 30 mL/min air flow with a rate of 10 °C/min. Mass spectral analysis of gaseous products released during the heating was performed using a QMS 403 C Aëolos quadrupole mass spectrometer (Netzsch, Germany). The study of paramagnetic centers was performed on a Bruker ELEXSYS-580 EPR spectrometer (Billerica, MA, USA) with a working frequency of 9.5 Hz and a sensitivity of 5 × 10^10^ spin/Gs. The *g*-values were determined based on Mn^++^ standard.

The oxidative surface-active sites were studied by the method of thermo-programmed reduction with hydrogen (TPR-H_2_) on the Chemisorb 2750 (Micromeritics, Norcross, GA, USA). The pre-treatment of the samples before the measurements was carried out in oxygen flow (20 mL/min) and included heating (10 °C/min) to 200 °C, annealing at 200 °C for 30 min, and cooling down to room temperature. During the TPR-H_2_ experiment, a H_2_/Ar gas mixture (8 vol.% H_2_) was passed through a flow-through quartz test tube with a sample. Heating (10 °C/min) was carried out to 900 °C (in the case of the SnSi19 sample to 1000 °C).

**Figure 1:**
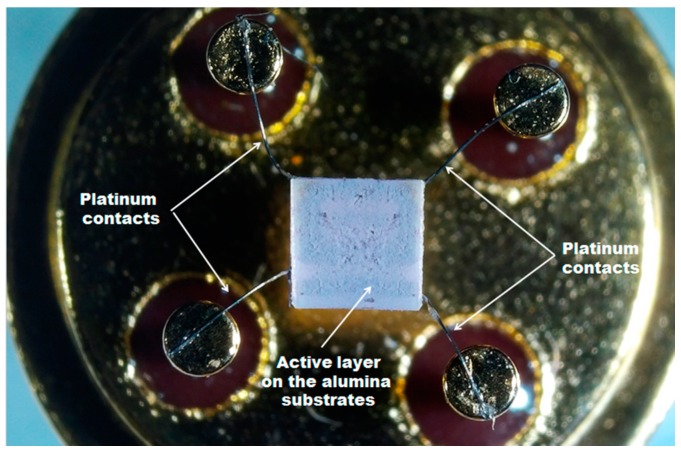
Active layer of SnO_2_/SiO_2_ nanocomposite on Al_2_O_3_ substrate fixed to the chip holder.

For electrophysical measurements, the powders of SnO_2_ and SnO_2_/SiO_2_ nanocomposites were mixed with α-terpineol (90%, Merck, Darmstadt, Germany) to form a paste and then deposited on alumina substrates with platinum contacts on the top side and a platinum heater on the back side. Thick films thus obtained were dried at 50 °C for 24 h and annealed at 300 °C using the back side heater (Figure 1). The registration of sample resistance was carried out automatically in the voltage stabilized DC mode with applied voltage of 1.3 V. The interaction of nanocomposites with oxygen was investigated *in situ* by measuring the conductivity of sensors depending on the oxygen partial pressure in the gas phase. To create gas mixtures with a pre-assigned oxygen content the commercially available Ar (no more than 0.002 vol. % O_2_) and synthetic air (20 vol. % O_2_) were used. In all experiments, the gas mixture flow was maintained constant at 100 ± 0.5 mL/min. Gas mixtures with fixed oxygen concentrations (0.002, 2, 5, 10, 15, and 20 vol.%) were prepared by mixing synthetic air and Ar using electronic gas flow controllers (Bronkhorst, Ruurlo, Netherlands). The measurements were carried out in the temperature range of 400–200°C. Between the temperature changes, the sensors were kept in Ar flow for 40 min.

## 3. Results and Discussion

**Figure 2:**
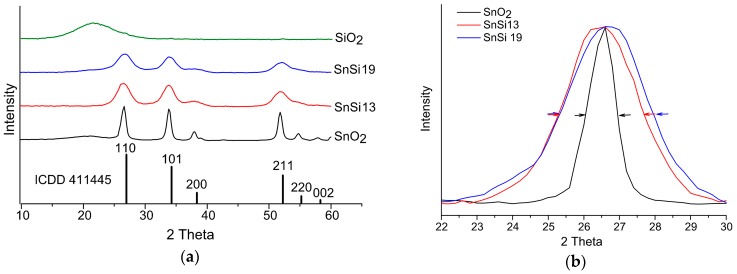
(**a**) Diffractograms of nanocrystalline SnO_2_, SiO_2_, and SnO_2_/SiO_2_ nanocomposites. (**b**) Normalized (110) diffraction peak of SnO_2_ phase in SnO_2_ and SnO_2_/SiO_2_ nanocomposites.

Energy dispersive X-ray spectroscopy (EDX) analysis of nanocomposites showed that their composition corresponds to that specified during synthesis (Table 3) [16]. X-ray diffraction revealed that SnO_2_ (cassiterite, ICDD 41-1445) is the only crystalline phase in all samples. Silicon oxide obtained under similar hydrothermal conditions in the absence of SnO_2_·xH_2_O xerogel is X-ray amorphous (Figure 2a). As evidenced by the increase in the width of SnO_2_ reflections (Figure 2b), the increase in silicon content in the nanocomposites leads to the decrease in the size of SnO_2_ crystallites under conditions of identical isothermal annealing. According to the low-temperature nitrogen adsorption data, the addition of SiO_2_ prevents sintering of tin dioxide particles during high-temperature annealing and allows obtaining samples with high specific surface area (Table 3).

**Figure 3:**
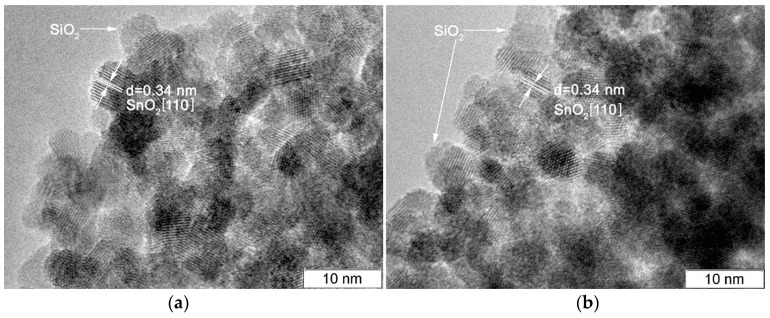
Images of: (**a**) SnSi 13; (**b**) SnSi 19 samples.

By HRTEM, it was found [16] that nanocrystalline SnO_2_ is formed by large crystalline nanoparticles, while SiO_2_ is completely amorphous. On the images of SnSi13 (Figure 3a) and SnSi19 (Figure 3b) samples, crystalline SnO_2_ particles (8–12 nm) and amorphous SiO_2_ particles (5–15 nm) that are distributed over the surface of the semiconductor oxide can be distinguished.

**Figure 4:**
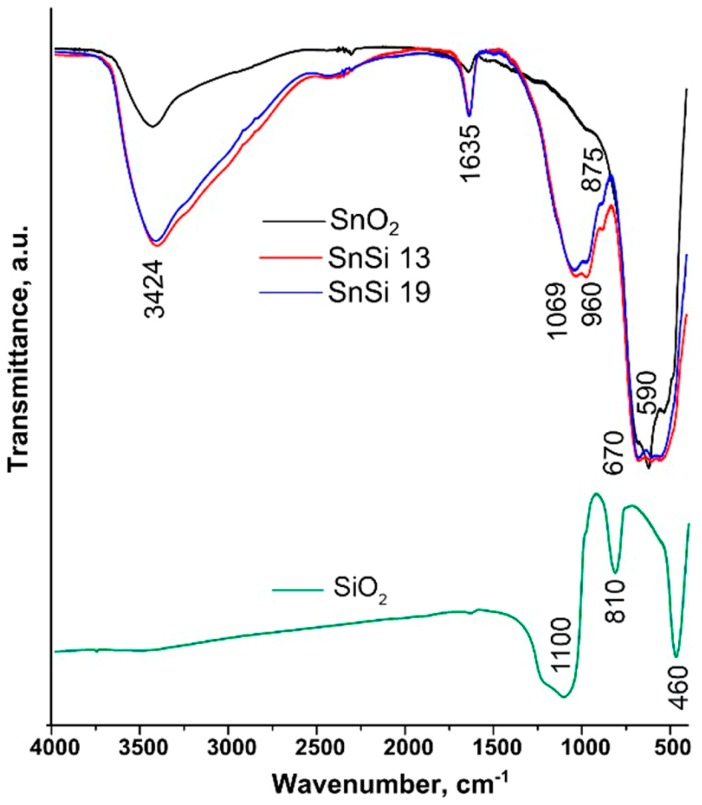
The IR spectra of the SnO_2_, SnSi13, SnSi19, and SiO_2_.

Using IR spectroscopy, it was studied how the addition of silicon dioxide affects the type and concentration of active groups on the SnO_2_ surface. The normalization of the IR spectra of composite samples to the intensity of Sn–O–Sn oscillations (670 cm^−1^) showed an increase in the concentration of hydroxyl groups on the surface of the samples with the growth of SiO_2_ content (Figure 4). In the range of 700–400 cm^−1^, the spectra of SnSi 13 and SnSi 19 contain the peaks corresponding to all the vibrations of individual SnO_2_ and SiO_2_. The detailed assignment [17,18,19] of the oscillations in IR spectra of nanocomposites is presented in Table 4.

**Figure 5:**
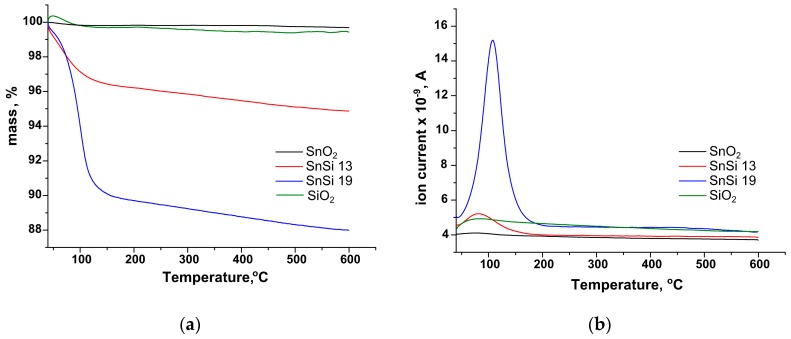
(**a**) TG curves; (**b**) temperature dependences of the H_2_O ion current (m/z = 18) for SnO_2_, SnSi 13, SnSi 19, and SiO_2_, kept in a desiccator at RH ≈ 100% for two days.

The observed trend to increase the number of hydroxyl groups on the surface of composite samples is in agreement with the results of the analysis of the amount of water desorbed from the surface of SnO_2_, SnSi 13, SnSi 19, and SiO_2_ samples. The study was carried out by thermogravimetric (TG) analysis, before which the samples were kept in a desiccator at RH ≈ 100% for two days. Based on the data obtained, it can be concluded that more water is desorbed from the surface of nanocomposites than from pure SnO_2_ and SiO_2_ (Figure 5, Table 5). Since this increase in adsorption capacity is characteristic of SnO_2_/SiO_2_ nanocomposites, it can be assumed that adsorption sites for water molecules are formed on the SnO_2_/SiO_2_ interface.

**Figure 6:**
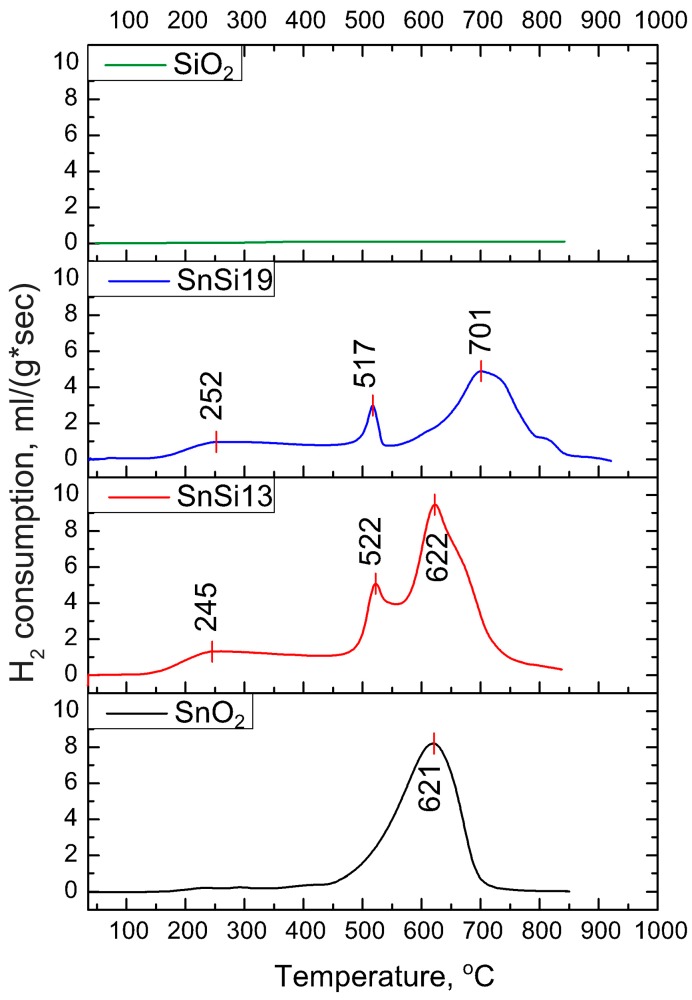
Profiles SnO_2_, SnSi 13, SnSi 19, and SiO_2_.

The concentration of surface oxygen containing species was estimated by the method of thermo-programmed reduction with hydrogen (TPR-H_2_). Figure 6 shows the temperature dependences of hydrogen consumption during the reduction of SnO_2_, SnSi 13, SnSi 19, and SiO_2_. In the experimental conditions, the reduction of pure silicon dioxide doesn’t occur. For SnO_2_ and SnO_2_/SiO_2_ nanocomposites, several regions can be distinguished in TPR profiles. The first peak is in the range of 200–300 °C, which corresponds to the reduction of chemisorbed oxygen (O_2_^−^, O^−^, O^2−^) and surface OH^−^ groups:

(1) $$O_{2\;\left(\mathrm{surf}\right)}+2H_{2\;\left(\mathrm{gas}\right)}\to 2H_{2}O_{\left(\mathrm{gas}\right)}$$

(2) $$O_{\left(\mathrm{surf}\right)}+H_{2\;\left(\mathrm{gas}\right)}\to H_{2}O_{\left(\mathrm{gas}\right)}$$

(3) $$2{\mathrm{OH}}_{\left(\mathrm{surf}\right)}+H_{2\;\left(\mathrm{gas}\right)}\to 2H_{2}O_{\left(\mathrm{gas}\right)}$$

On the SnO_2_ TPR profile, a peak with a maximum at 621 °C corresponds to the reduction of SnO_2_ to metallic tin:

(4) $${\mathrm{SnO}}_{2}+2H_{2\;\left(\mathrm{gas}\right)}\to \mathrm{Sn}+2H_{2}O_{\left(\mathrm{gas}\right)}$$

In the case of composite samples, two peaks appear in this temperature region. The appearance of a signal with a maximum in the region of 520 °C is possibly due to the partial reduction of Sn^4+^ → Sn^2+^ [19,20]:

(5) $${\mathrm{SnO}}_{2}+H_{2\;\left(\mathrm{gas}\right)}\to \mathrm{SnO}+2H_{2}O_{\left(\mathrm{gas}\right)}$$

The peak corresponding to the Sn^4+^ → Sn^0^ reduction for the SnSi 19 sample is shifted toward higher temperatures with a maximum of 701 °C. This may be due to the difficult reduction of tin atoms linked with SiO_4_ groups.

The results of the TPR-H_2_ experiments are summarized in Table 6. During the measurements, the signal from the thermal conductivity detector (TCD, arb. units), which is proportional to the rate of hydrogen consumption, was registered depending on the temperature inside the reactor. The quantity of hydrogen consumed in a given temperature range (25–400 °C or 400–900 °C) was calculated using calibration curves obtained for a reference Ag_2_O sample. The total quantity of hydrogen consumed during the experiment (Table 6) for all the samples varies from 2.0 to 2.8 mol H_2_ per mol SnO_2._ The amount of hydrogen consumed during SnO_2_ reduction for SnO_2_ and SnSi13 samples (temperature range 400–900 °C) is *n* = 2.1–2.3 mol H_2_ per 1 mol SnO_2_ (Table 6), which is close to the theoretical value *n* = 2, corresponding to the reduction of tin dioxide to the metal tin (reaction (4)). An increase in the silicon content leads to a significant reduction in the amount of hydrogen consumed in this temperature range (*n* = 1.5 mol H_2_ per 1 mol SnO_2_ for SnSi 19 nanocomposite). This may be due to the fact that some Sn cations bonded to SiO_4_ groups cannot be completely reduced to Sn^0^ under experimental conditions. Compared with the nanocrystalline SnO_2_, in the case of reduction of nanocomposites, an increase in the amount of hydrogen consumed in the low-temperature range (25–400 °C) is observed (Table 6). This is due to an increase in the quantity of surface oxygen-containing species (chemisorbed oxygen and hydroxyl groups), caused by a reduced SnO_2_ crystallite size and increased specific surface area of the nanocomposites compared with unmodified SnO_2_. 

**Figure 7:**
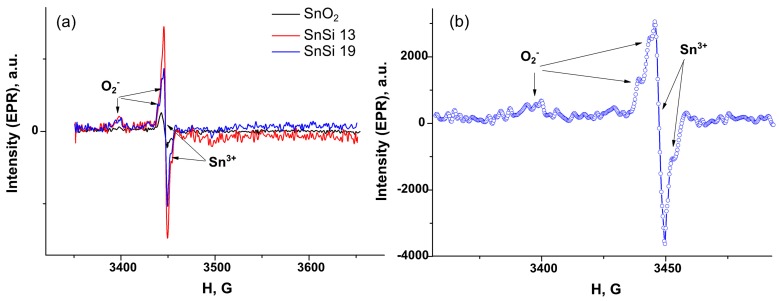
(**a**) Electron-paramagnetic resonance (EPR) spectra of SnO_2_ samples and SnSi 13, SnSi19 composites; (**b**) EPR spectrum of the SnSi19 sample in a narrow magnetic field range.

The obtained samples were studied by EPR spectroscopy to assess the effect of SiO_2_ on the concentration of paramagnetic centers in tin dioxide. In the spectra obtained, the EPR signal has a complex shape and is a superposition of several lines. As the analysis showed, the spectrum consists of two EPR signals, characterized by the following values of g-factors: (I) g_1_ = 2.027, g_2_ = 2.008, g_1_ = 2.003 in the magnetic field range ΔH = 3350–3440 G and (II) g_1_ = 1.9989, g_2_ = 1.9981 in the magnetic field range ΔH = 3440–3480 G (Figure 7a,b). According to the literature, the first of the detected EPR signals, characterized by orthorhombic symmetry, can be attributed to the oxygen anion radicals O_2_^-^ [21]. The second EPR signal, characterized by a symmetry close to axial, belongs to the Sn^3+^ paramagnetic centers [22,23]. Perhaps the presence of Sn^3+^ centers is due to the charge transfer from hydroxyl groups to Sn^4+^ ions. The calculated concentrations of paramagnetic centers Ns(Sn^3+^) and Ns(O_2_^−^) are given in Table 7. The obtained values were assigned to the SnO_2_ mass fraction in SnO_2_/SiO_2_ nanocomposites. With an increase in the SiO_2_ content, a non-monotonic increase in the number of O_2_^−^ and Sn^3+^ centers is observed.

The set of the obtained results allows us to conclude that the introduction of silicon dioxide during hydrothermal treatment of amorphous xerogel SnO_2_·xH_2_O and subsequent high-temperature annealing leads to the significant increase in the amount of oxygen-containing surface species, namely chemisorbed oxygen and hydroxyl groups, as well as an increase in the number of paramagnetic centers Sn^3+^, in which tin is in a low oxidation state.

Chemisorption of oxygen occurs on the surface of semiconductor materials with electron capture, thereby affecting the conductivity of the semiconductor:

(6) $${\;O}_{2\;\left(\mathrm{ads}.\right)}\overset{e^{-}}{\to }O_{2\left(\mathrm{ads}.\right)}^{-}\overset{e^{-}}{\to }2O_{\left(\mathrm{ads}.\right)}^{-}\overset{2e^{-}}{\to }2O_{\left(\mathrm{lattice}\right)}^{2-}.$$

**Figure 8:**
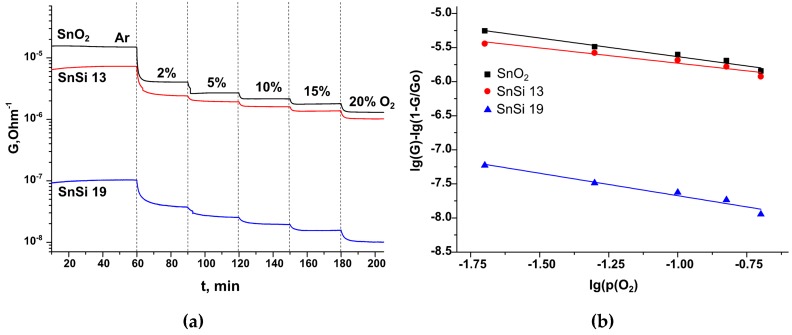
(**a**) Dependencies of the conductivity of samples on the O_2_ partial pressure f at 400 ^o^C; (**b**) Dependencies $\mathrm{lg}\left(G\right)-\mathrm{lg}\left(1-\frac{G}{G_{0}}\right)$ vs. $\mathrm{lg}\left(p_{O_{2}}\right)$ for SnO_2_, SnSi 13, and SnSi 19 samples.

The ionized forms of chemisorbed oxygen are the main active groups on the surface of SnO_2_, interacting with the target reducing gas. Surface reactions leading to the formation of sensor response, in general, can be written as:

(7) $$2R_{\left(\mathrm{gas}\right)}+O_{2\left(\mathrm{ads}\right)}^{-}\to 2{\mathrm{RO}}_{\left(\mathrm{gas}\right)}+e^{-}$$

(8) $$R_{\left(\mathrm{gas}\right)}+O_{\left(\mathrm{ads}\right)}^{-}\to {\mathrm{RO}}_{\left(\mathrm{gas}\right)}+e^{-}$$

where R is a reducing gas molecule and RO is the product of oxidation of R by chemisorbed oxygen. The predominant form of chemisorbed oxygen on the SnO_2_ surface is determined by the measurement temperature, the size of the SnO_2_ crystallites, and the presence of modifiers on their surface [6,24,25]. To estimate the predominant form of chemisorbed oxygen on the surface of SnO_2_ and SnO_2_/SiO_2_ nanocomposites, the *in situ* measurements of electrical conductivity, depending on the oxygen partial pressure in the gas phase, were carried out. As the partial pressure of O_2_ in the gas phase increases, the conductivity of all samples decreases (Figure 8a), which is typical for *n*-type semiconductor oxides. The conductivity is reduced by the reaction occurring on the surface of the samples during oxygen chemisorption [24,26]:

(9) $$\beta /2O_{2\left(\mathrm{gas}\right)}+{\alpha e}^{-}=O_{\beta \left(\mathrm{ads}.\right)}^{\alpha -}$$

where O_2_ gas is an oxygen molecule in the ambient atmosphere, O^α-^_β(ads.)_ is a chemisorbed oxygen species with: α = 1 for singly ionized forms, α = 2 for doubly ionized forms, β = 1 for atomic forms, and β = 2 for molecular forms. According to the mass action law, in the steady state, the concentration of electrons capable of reaching the surface (n_s_) is determined by the partial pressure of gas p(O_2_) and the type of chemisorbate (parameters α, β):

(10) $$n_{s}^{\alpha }=k_{\mathrm{des}.}/k_{\mathrm{ads}.}\theta p{\left(O_{2}\right)}^{-\beta /2}$$

where k_ads_ and k_des_ are adsorption and desorption constants, respectively, and θ is the part of filled adsorption sites. For a porous nanocrystalline layer, the electrical conductivity (G) linearly depends on p(O_2_) in logarithmic coordinates:

(11) $$\mathrm{lg}\left(G\right)-\mathrm{lg}\left(1-\frac{G}{G_{0}}\right)=const-m\cdot lg\left(p_{O_{2}}\right)$$

where G is conductivity in the presence of oxygen and G_0_ is conductivity in an inert atmosphere (argon) [24]. The parameter m = β/2α corresponds to the form of chemisorbed oxygen. Depending on temperature and grain size, the predominant form of chemisorbed oxygen on the surface of *n*-type semiconductor oxides can be $O_{2}^{-}$ (m = 1), $O^{-}$ (m = 0.5) or $O^{2-}$ (m = 0.25) [24,26].

Based on the data obtained, the dependencies of $\mathrm{lg}\left(G\right)-\mathrm{lg}\left(1-\frac{G}{G_{0}}\right)$ vs. $\mathrm{lg}\left(p_{O_{2}}\right)$ were plotted (Figure 8b). Linearization in these coordinates is valid for nanoparticles smaller than 25 nm [24,25,26]. The values of the coefficient *m*, corresponding to the predominant type of chemisorbed oxygen, were calculated from the slope of the obtained dependences. The results are presented in Table 8.

The error values of the coefficients m for the measurements effectuated at 200 and 300 °C are too large for accurate identification of the predominant form of chemisorbed oxygen. However, by analyzing the data presented in Table 8, the following trends can be identified: (i) At 400 °C, the values of the coefficient *m* for SnO_2_ and SnSi 13 coincide within the error and correspond to the predominant form of chemisorbed oxygen $O^{-}$. For the SnSi 19 nanocomposite, the value of the coefficient *m* corresponds to the simultaneous presence of atomic $O^{-}$ and molecular $O_{2}^{-}$ forms of chemisorbed oxygen; (ii) with a decrease in the measurements temperature, an increase in the coefficient *m* is observed, which corresponds with an increase in the proportion of chemisorbed oxygen in the $O_{2}^{-}$ form; (iii) in general, an increase in the silicon content in nanocomposites leads to an increase in the contribution of molecular ions $O_{2}^{-}$, which is consistent with the data obtained by EPR spectroscopy.

**Figure 9:**
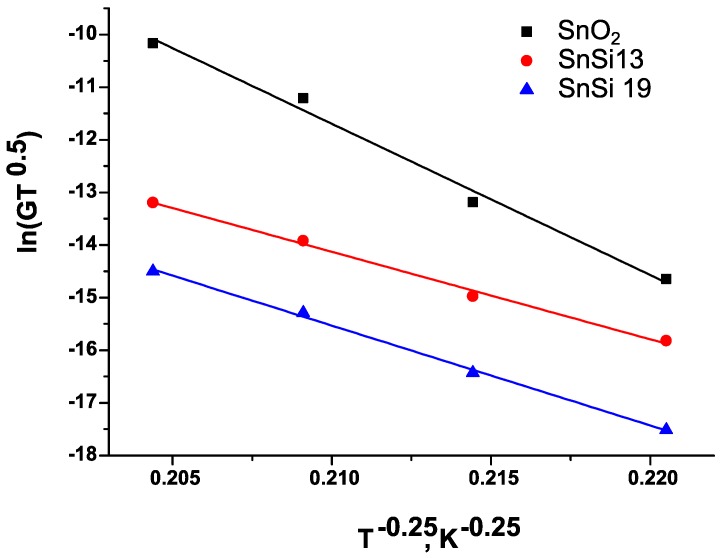
The thermal dependences of the conductivity SnO_2_, SnSi 13, and SnSi 19 samples in Mott coordinates in the temperature range 400–150 °C.

A change in the type and concentration of charged active centers affects the electrical conductivity of nanocrystalline semiconductors. As it was demonstrated by impedance spectroscopy [27], the transport properties of nanocrystalline SnO_2_ are dominated by hopping conduction through disordered crystallite boundaries. The obtained temperature dependences of conductivity are well straightened in Mott coordinates (Figure 9). 

In this model, the expression for conductivity (G) is written as:

(12) $$G=\;\frac{G_{M}}{T^{0.5}}\exp\left[-{\left(\frac{T_{M}}{T}\right)}^{0.25}\right],\;$$

where G_M_ and T_M_ are characteristic Mott parameters. The coefficient G_M_ is the conductivity of the film at an inverse temperature of 1/T, tending to 0. As a result of the logarithm of Equation (12), we obtain:

(13) $$\ln\left(G\cdot T^{0.5}\right)=\ln\left(G_{M}\right)-{\left(\frac{T_{M}}{T}\right)}^{0.25}.$$

when linearizing the dependence $\ln\left(G\cdot T^{0.5}\right)=f\left(T^{-0.25}\right)$, the T_M_ value can be calculated from the slope of the straight line. The parameter T_M_ is inversely related to the density of localized states near the Fermi level N(E_F_):

(14) $$T_{M}=\frac{16\alpha^{3}}{k_{B}N\left(E_{F}\right)},$$

where α is the value describing the degree of spatial localization of the wave function and k_B_ is the Boltzmann constant. Knowing the of N(E_F_) value, one can calculate the hopping distance R_hop_:

(15) $$R_{\mathrm{hop}}={\left(\frac{9}{8{\pi \alpha k}_{B}\mathrm{TN}\left(E_{F}\right)}\right)}^{0.25}$$

and hopping energy W_hop_:

(16) $$W_{\mathrm{hop}}=\frac{3}{4{\pi R}^{3}N\left(E_{F}\right)}.$$

Table 9 shows the parameters characterizing the conductivity of the samples under study in the framework of the Mott model. In the calculations, the value of α was taken equal to 1.24 nm^−1^ [28].

The data obtained satisfies the criteria of applicability of the Mott model. For all the cases under consideration, the conditions W > kT and αR >> 1 are satisfied [28]. The obtained values of the Mott parameters indicate a high degree of disorder of the studied systems. Linearization of experimental data in Mott coordinates (Figure 9) indicates that the charge transfer in nanocrystalline SnO_2_ and nanocomposites is carried out by the hopping conductivity of electrons through localized states lying near the Fermi level. The addition of SiO_2_ leads to a decrease in the slope of the linear dependences $\ln\left(G\cdot T^{0.5}\right)=f\left(T^{-0.25}\right)$: T_M_(SnO_2_) > T_M_(SnSi 19) > T_M_(SnSi 13), which indicates an increase in the density of unfilled local states and is consistent with data obtained by EPR spectroscopy. Compared to nanocrystalline SnO_2_, an increase in the concentration of Sn^3+^ in SnO_2_/SiO_2_ nanocomposites also causes a decrease in the hopping distance R_hop_ and hopping energy W_hop_. This should lead to an increase in the mobility of charge carriers in nanocomposites. The observed decrease in the electrical conductivity of materials with an increase in the SiO_2_ concentration in nanocomposites is apparently due to a decrease in the concentration of charge carriers because of their localization on chemisorbed oxygen (reaction (9)), which amount increases in a row: SnO_2_ < SnSi 13 < SnSi 19 (Table 7). 

## 4. Conclusions

Nanocomposites SnO_2_/SiO_2_ were synthesized via a hydrothermal route. The introduction of silicon dioxide at the stage of hydrothermal treatment of β-stannic acid allows obtaining semiconductor materials with a high specific surface area resistant to sintering at 600 °C. The modification of SnO_2_ nanocrystalline matrix with amorphous SiO_2_ results in the increase in the concentration of paramagnetic centers Sn^3+^_,_ surface hydroxyl groups and chemisorbed oxygen and leads to a decrease in the negative charge on chemisorbed oxygen species. The conductivity of nanocomposites is described in the framework of the Mott hopping conduction model. Compared to nanocrystalline SnO_2_, an increase in the concentration of Sn^3+^ in SnO_2_/SiO_2_ nanocomposites causes a decrease in the hopping distance and hopping energy, which should lead to an increase in the mobility of charge carriers in the nanocomposites.

## Figures and Tables

**Table 1:** Characteristics and gas sensor properties of SnO_2_/SiO_2_ nanocrystalline materials.

Material Type	Synthesis Method	$\frac{\left[Si\right]}{\left[Si\right]+\left[Sn\right]}\;\mathrm{mol}.\%$	Gas	C_gas_, ppm	T_mes_, °C	Sensor Signal	Reference
Thick film	Direct flame spray pyrolysis	7.8	Ethanol	50	320	318	[9]
Core–shell nanofibers	Single-spinneret electrospinning	75	Ethanol	200	not defined	37	[11]
Powders	Wet-chemical modification through the dehydration-condensation reaction	4.8	CO	100	260	350	[12]
Powders	Micro-emulsion followed by ultrasonic-assisted deposition-precipitation method	33	Ethanol Acetone	300 300	270 270	1066 2193	[13]
Thin film	Sol-gel method and electron-beam irradiation treatment	20	Acetone Isopropanol	1000	300	27 16	[14]
Core–shell nanofibers	Template synthesis	not defined	H_2_ CO	200 200	450 400	500 100	[15]

**Table 2:** Sensor properties of nanocrystalline SnO_2_ and SnO_2_/SiO_2_ nanocomposites in CO detection [16].

Sample	$\frac{\left[Si\right]}{\left[Si\right]+\left[Sn\right]}\mathrm{mol}.\%{}^{\left(a\right)}$	R (400 °C), Ω^(*b*)^	LDL, ppm*^(c)^*		$\tau_{res}^{90},\;\min{}^{\left(d\right)}$		$\tau_{rec}^{90},\;\min{}^{\left(e\right)}$	
			RH = 1%	RH = 20%	RH = 1%	RH = 20%	RH = 1%	RH = 20%
SnO_2_	0	7.3 × 10^7^	1.5	9.0	3.6 ± 0.2	5.2 ± 0.6	6.4 ± 0.3	5.8 ± 0.7
SnSi13	13	3.2 × 10^8^	4.3	7.0	2.7 ± 0.1	2.6 ± 0.1	7.2 ± 0.1	7.0 ± 0.7
SnSi19	19	2.2 × 10^9^	3.3	7.8	2.6 ± 0.1	2.9 ± 0.1	7.9 ± 0.4	6.8 ± 0.8

*^(a)^*determined by energy dispersive X-ray spectroscopy (EDX); *^(b)^*resistance in dry air at 350 °C; *^(c)^*CO low detection limit; *^(d)^*90% response time at the temperature of maximum sensor signal (100 ppm CO); *^(e)^*90% recovery time at the temperature of maximum sensor signal (100 ppm CO).

**Table 3:** Composition and microstructure parameters of the SnO_2_ and SnO_2_/SiO_2_ nanocomposites.

Sample	$\frac{\left[Si\right]}{\left[Si\right]+\left[Sn\right]}\;\mathrm{mol}.\%{}^{\left(a\right)}$	*d_XRD_*(SnO_2_), nm*^(b)^*	*S_BET_* ± 5 m^2^/g*^(c)^*
SnO_2_	0	11 ± 1	23
SnSi13	13	7 ± 1	99
SnSi19	19	6 ± 1	156
SiO_2_	100	-	327

*^(a)^*determined by EDX; *^(b)^*estimated using the Scherer formula; *^(c)^*determined by low-temperature N_2_ adsorption.

**Table 4:** Assignment of the oscillations present in the IR spectra of SnO_2_, SnSi 13, SnSi 19, and SiO_2_.

Wavenumber, cm^−1^	Oscillation	Ref.
3650–2500	ν(O-H)	[17]
1635	δ(H_2_O)	[17]
1250–870	ν_ass_(Si–O–Si), ν(Si–OH)	[18]
960	ν_ass_(O_3_Si–Sn)	[19]
810	ν_sim_(Si–O–Si)	[18]
670	ν_ass_(Sn–O–Sn)	[18]
590	ν(Sn–OH)	[17]
530	ν_sim_(Sn–O)	[17]
460	δ(Si–O)	[18]

**Table 5:** Estimation of the amount of desorbed water according to the results of thermal analysis.

Sample	Amount of Desorbed Water, mol/m^2^
SnO_2_	4.9 × 10^−6^
SnSi13	1.9 × 10^−5^
SnSi19	3.4 × 10^−5^
SiO_2_	5.3 × 10^−7^

**Table 6:** The results of the thermo-programmed reduction with hydrogen (TPR-H_2_) experiments.

Sample	Hydrogen Consumption, mol H_2_ per 1 mol SnO_2_		
	Total	at 25–400 °C	at 400–900 °C
SnO_2_	2.2 ± 0.3	0.1 ± 0.03	2.1 ± 0.3
SnSi 13	2.8 ± 0.3	0.5 ± 0.1	2.3 ± 0.3
SnSi 19	2.0 ± 0.2	0.5 ± 0.1	1.5 ± 0.2

**Table 7:** Concentration of paramagnetic centers in SnO_2_ and SnO_2_/SiO_2_ nanocomposites.

Sample	Ns(O_2_^−^), g^−1^ SnO_2_	Ns(Sn^3+^), g^−1^ SnO_2_
SnO_2_	3.0 × 10^13^	1.3 × 10^14^
SnSi 13	9.0 × 10^13^	8.8 × 10^14^
SnSi 19	1.2 × 10^14^	5.8 × 10^14^

**Table 8:** Coefficient *m* (Equation (11)) obtained from $\mathrm{lg}\left(G\right)-\mathrm{lg}\left(1-\frac{G}{G_{0}}\right)$ vs. $\mathrm{lg}\left(p_{O_{2}}\right)$ dependencies.

Sample	Coefficient m		
	400 °C	300 °C	200 °C
SnO_2_	0.55 ± 0.05	0.51 ± 0.08	-
SnSi 13	0.46 ± 0.06	0.70 ± 0.20	0.60 ± 0.20
SnSi 19	0.67 ± 0.08	0.60 ± 0.10	0.80 ± 0.30

**Table 9:** Parameters calculated within the Mott conductivity model: T_M_, N(E_F_), R_hop_, and W_hop_ for samples SnO_2_, SnSi 13, and SnSi 19.

Sample.	T_M_, 10^8^ K	N(E_F_), 10^17^ eV^−1^·cm^−3^	R_hop_, nm		W_hop_, eV	
			25 °C	200 °C	25 °C	200 °C
SnO_2_	68.9	0.52	21	19	0.46	0.67
SnSi 13	7.8	4.7	12	11	0.27	0.39
SnSi 19	13.5	2.6	14	13	0.31	0.43

## References

[1] Joshi N., Hayasaka T., Liu Y., Liu H., Oliveira O.N., Lin L. (2018). A review on chemiresistive room temperature gas sensors based on metal oxide nanostructures, graphene and 2D transition metal dichalcogenides. Microchim. Acta.

[2] Liu X., Ma T., Pinna N., Zhang J. (2017). Two-Dimensional Nanostructured Materials for Gas Sensing. Adv. Funct. Mater..

[3] Zhang J., Liu X., Neri G., Pinna N. (2016). Nanostructured Materials for Room-Temperature Gas Sensors. Adv. Mater..

[4] Das S., Jayaraman V. (2014). SnO_2_: A comprehensive review on structures and gas sensors. Prog. Mater. Sci..

[5] Korotcenkov G. (2013). Handbook of Gas Sensor Materials: Properties, Advantages and Shortcomings for Applications. Integrated Analytical Systems.

[6] Krivetskiy V.V., Rumyantseva M.N., Gaskov A.M. (2013). Chemical modification of nanocrystalline tin dioxide for selective gas sensors. Russ. Chem. Rev..

[7] Marikutsa A.V., Vorobyeva N.A., Rumyantseva M.N., Gaskov A.M. (2017). Active sites on the surface of nanocrystalline semiconductor oxides ZnO and SnO_2_ and gas sensitivity. Russ. Chem. Bull..

[8] Krivetskiy V., Efitorov A., Arkhipenko A., Vladimirova S., Rumyantseva M., Dolenko S., Gaskov A. (2018). Selective detection of individual gases and CO/H_2_ mixture at low concentrations in air by single semiconductor metal oxide sensors working in dynamic temperature mode. Sens. Actuators B.

[9] Tricoli A., Graf M., Pratsinis S.E. (2008). Optimal Doping for Enhanced SnO_2_ Sensitivity and Thermal Stability. Adv. Funct. Mater..

[10] Tricoli A. (2012). Structural Stability and Performance of Noble Metal-Free SnO_2_-Based Gas Sensors. Biosensors.

[11] Liu Y., Yang P., Li J., Matras-Postolek K., Yue Y., Huang B. (2016). Formation of SiO_2_@SnO_2_ Core-Shell Nanofibers and Their Gas Sensing Properties. RSC Adv..

[12] Zhan Z., Chen J., Guan S., Si L., Zhang P. (2013). Highly Sensitive and Thermal Stable CO Gas Sensor Based on SnO_2_ Modified by SiO_2_. J. Nanosci. Nanotechnol..

[13] Asgari M., Saboor F.H., Mortazavi Y., Khodadadi A.A. (2017). SnO_2_ decorated SiO_2_ chemical sensors: Enhanced sensing performance toward ethanol and acetone. Mater. Sci. Semicond. Process..

[14] Nalimova S.S., Myakin S.V., Moshnikov V.A. (2016). Controlling Surface Functional Composition and Improving the Gas-Sensing Properties of Metal Oxide Sensors by Electron Beam Processing. Glass Phys. Chem..

[15] Gunji S., Jukei M., Shimotsuma Y., Miura K., Suematsu K., Watanabe K., Shimanoe K. (2017). Unexpected gas sensing property of SiO_2_/SnO_2_ core-shell nanofibers in dry and humid conditions. J. Mater. Chem. C.

[16] Gulevich D., Rumyantseva M., Gerasimov E., Marikutsa A., Krivetskiy V., Shatalova T., Khmelevsky N., Gaskov A. (2019). Nanocomposites SnO_2_/SiO_2_ for CO gas sensors: Microstructure and reactivity in the interaction with the gas phase. Materials.

[17] Nakamoto K. (1997). Infrared and Raman Spectra of Inorganic and Coordination Compounds.

[18] Ferreira C.S., Santos P.L., Bonacin J.A., Passos R.R., Pocrifka L.A. (2015). Rice Husk Reuse in the Preparation of SnO_2_/SiO_2_Nanocomposite. Mater. Res..

[19] Park P.W., Kung H.H., Kim D.-W., Kung M.C. (1999). Characterization of SnO_2_/Al_2_O_3_ Lean NO_x_ Catalysts. J. Catal..

[20] Ho S.-T., Dinh Q.-K., Tran T.-H., Nguyen H.-P., Nguyen T.-D. (2013). One-Step Synthesis of Ordered Sn-Substituted SBA-16 Mesoporous Materials Using Prepared Silica Source of Rice Husk and Their Selectively Catalytic Activity. J. Can. Chem. Eng..

[21] Gurlo A. (2006). Interplay between O_2_ and SnO_2_: Oxygen Ionosorption and Spectroscopic Evidence for Adsorbed Oxygen. Chem. Phys. Chem..

[22] Chiodini N., Ghidini S., Paleari A. (2001). Mechanisms responsible for the ultraviolet photosensitivity of SnO_2_-doped silica. Phys. Rev. B..

[23] Chiodini N., Meinardi F., Morazzoni F., Padovani J., Paleari A., Scotti R., Spinolo G. (2001). Thermally induced segregation of SnO_2_ nanoclusters in Sn-doped silica glasses from over saturated Sn-doped silica xerogels. J. Mater. Chem..

[24] Rumyantseva M.N., Makeeva E.A., Badalyan S.M., Zhukova A.A. (2009). Nanocrystalline SnO_2_ and In_2_O_3_ as Materials for Gas Sensors: The Relationship between Microstructure and Oxygen Chemisorption. Thin Solid Films.

[25] Zaretskiy N.P., Menshikov L.I., Vasiliev A.A. (2012). On the origin of sensing properties of the nanostructured layers of semiconducting metal oxide materials. Sens. Actuators B..

[26] Barsan N., Weimar U. (2001). Conduction model of metal oxide gas sensors. J. Electroceram..

[27] Chizhov A.S., Rumyantseva M.N., Gaskov A.M. (2013). Frequency Dependent Electrical Conductivity of Nanocrystalline SnO_2_. Inorg. Mater..

[28] Mott N.F., Devis E.A. (1979). Electron Processes in Non-Crystalline Materials.

